# Anthropometric status of nurses working at a private hospital in Pietermaritzburg, KwaZulu-Natal

**DOI:** 10.4102/hsag.v27i0.1940

**Published:** 2022-11-21

**Authors:** Leah B. Yegambaram, Kirthee Pillay

**Affiliations:** 1 Department of Dietetics and Human Nutrition, College of Agriculture, Engineering and Science, University of KwaZulu-Natal, Pietermaritzburg, South Africa

**Keywords:** anthropometric status, nurses, overweight, obesity, body mass index, waist circumference, private hospital, KwaZulu-Natal

## Abstract

**Background:**

The prevalence of overweight and obesity among nurses in South Africa (SA) is increasing. This is a concern as overweight and obesity increase the risk for non-communicable diseases (NCDs).

**Aim:**

This study aimed to determine the anthropometric status of nurses working at a private hospital and to identify the factors related to a high body mass index (BMI).

**Setting:**

This study was conducted at a private hospital in Pietermaritzburg (PMB), KwaZulu-Natal (KZN).

**Methods:**

Weight, height and waist circumference (WC) measurements were taken, using standardised procedures. A self-administered questionnaire was developed to collect data on factors associated with a high BMI.

**Results:**

Most participants were overweight or obese (86.2%; *n* = 112). The mean BMI of females (33.55 kg/m^2^) was significantly higher than that of males (28.08 kg/m^2^) (*p* = 0.043). Nurses who did not smoke had a significantly higher BMI (33.84 kg/m^2^) than those who smoked (29.58 kg/m^2^) (*p* = 0.030). Nurses who skipped meals had a higher mean BMI (33.75 kg/m^2^) than those who did not skip meals (29.63 kg/m^2^) (*p* = 0.005). Most females had a WC above 88 cm (66.2%; *n* = 86), indicating a substantially increased risk for metabolic complications.

**Conclusion:**

There was a high prevalence of overweight and obesity among the nurses according to BMI. According to WC, most female nurses had an increased risk for metabolic complications. Being female, not smoking and skipping meals were associated with a higher BMI.

**Contribution:**

This study highlights the increasing prevalence of overweight and obesity among nurses.

## Introduction

According to the World Health Organization (WHO), the global prevalence of obesity has tripled since 1975 (WHO 2021a). In 2016, more than 1.9 billion adults were overweight, with over 650 million of these adults classified as obese (WHO 2021a). The South African National Health and Nutrition Survey (SANHANES-1) reported that 20.1% and 24.8% of men and women were overweight, respectively, while 10.6% and 39.2%, respectively, were obese (Shisana et al. 2013). This indicates that obesity is more common among South African women than men (Shisana et al. 2013). The South African Demographic and Health Survey (SADHS) of 2016 found that 41% of women and 11% of men aged 15 years and older were obese (National Department of Health [NDoH] et al. 2019).

An accumulation of excess fat in the body over time results in overweight and obesity (WHO 2021a). Body mass index (BMI) is used to classify the degree of overweight and obesity in adults (WHO 2021a). It is calculated for an individual by dividing their weight in kilograms by their height in metres squared and is reported in kg/m^2^ (WHO 2021a). A BMI greater than or equal to 25 kg/m^2^ is classified as overweight, while a BMI greater than or equal to 30 kg/m^2^ is classified as obese (WHO 2021a). A raised BMI is a major risk factor for non-communicable diseases (NCDs) (WHO 2021a). Non-communicable diseases, also known as chronic diseases of lifestyle result from a combination of genetic, physiological, environmental and behavioural factors (WHO 2021b). The main NCDs include cardiovascular disease (CVD), cancer, chronic respiratory disease and diabetes (WHO 2021b).

Although BMI has traditionally been used to diagnose overweight, waist circumference (WC) may be a better predictor of CVD risk as it reflects abdominal obesity (WHO 2011). Increased levels of visceral adipose tissue are associated with several metabolic abnormalities, which increase the risk for type 2 diabetes mellitus and CVD (WHO 2011). In men and women, a WC greater than 94 cm and 80 cm, respectively, indicates an increased risk for metabolic complications, while a WC greater than 102 cm for men and 88 cm for women indicates a substantially increased risk for metabolic complications (WHO 2011).

Many studies have reported a high prevalence of overweight and obesity among nurses Goon et al. 2013; Kyle et al. 2017; Perry et al. 2018; Monakali et al. 2019; Sharif 2020. Overweight and obesity in nurses can result in poorer health, reduced productivity at work and ineffective patient care (Chen, Lim & Ivy 2021). A study conducted in the Eastern Cape, South Africa (SA) found that 76% of the professional nurses were obese, while 18% were overweight. The high prevalence of obesity in this study could be attributed to the work setting in primary healthcare facilities, where professional nurses are in a sitting position for many hours during consultations (Monakali et al. 2019). Another South African study found that 27.5% and 44.4% of nurses were overweight and obese, respectively. Factors contributing to the high prevalence of overweight and obesity among these nurses included a high energy intake, low physical activity and not knowing the health consequences of overweight and obesity (Goon et al. 2013). A study on nurses in Ghana found that 55.9% were obese with a higher obesity prevalence among female nurses with a higher professional ranking (Obirikorang et al. 2016). Consuming meals late at night and snacking in between meals were the most important lifestyle factors contributing to the increased obesity prevalence (Obirikorang et al. 2016). Other studies have also reported a higher mean BMI among female nurses, compared with males (Aryee et al. 2013; Monakali et al. 2019). Factors contributing to overweight and obesity among the nurses included low physical activity levels, skipping meals, being older and married (Aryee et al. 2013). Shift work among nurses, particularly working the night shift, may play a significant role in the development of obesity among nurses (Zhang et al. 2020).

Being overweight and obese and living with NCDs such as hypertension and diabetes were concerns reported by nurses working in the Western Cape Metropole, SA (Phiri et al. 2014). Nurses felt that being overweight impacted negatively on work performance. Nurses also reported that some of their overweight colleagues experienced difficulty in coping with their job demands. Consuming meals late, eating during stressful periods, low physical activity levels and working shifts all contributed to overweight and obesity among nurses (Phiri et al. 2014). Simfukwe, Van Wyk and Swart (2017) who conducted a study in three selected hospitals in Pietermaritzburg (PMB) found that although healthcare workers had some knowledge of obesity, they still consumed unhealthy meals because of convenience and the lack of healthy food options at the hospital. Study participants identified the high cost of healthy food as a barrier to healthy eating (Phiri et al. 2014; Simfukwe et al. 2017). A systematic review and meta-analysis by Chen et al. (2021) concluded that shift work, age, night shift work, sex, marital status, hours worked per week, stress levels and educational level of nurses were correlated to overweight and obesity in nurses.

It is important to assess the anthropometric status of nurses in order to identify those who are overweight and obese. Identifying the factors that are associated with a higher BMI among nurses can help to target interventions to address overweight and obesity among nurses. Addressing overweight and obesity among nurses may result in nurses being more physically active at work, having a lower risk for chronic diseases of lifestyle and being more confident when delivering health education to their patients on weight management. There is a paucity of published studies on the anthropometric status of nurses in PMB, KwaZulu-Natal (KZN). Therefore, this study aimed to determine the anthropometric status of nurses working at a private hospital in PMB, KZN and to identify the factors related to a high BMI (Yegambaram 2021).

## Research methods and design

### Study design

A quantitative, cross-sectional, descriptive study was conducted (Yegambaram 2021).

### Study population

The study population consisted of professional nurses, staff nurses and auxiliary nurses employed at a private hospital in PMB, KZN, SA. Four private hospitals in PMB were invited to participate in the study; however, only one hospital agreed to participate. Only private hospitals were included in the study as nurses in private hospitals do not have free access to an occupational health doctor, compared with nurses working at a public hospital. Nurses were selected to participate in the study as they usually spend the most time with patients, have an important role to play in promoting good health and are regarded as role models for their patients (Yegambaram 2021). However, they have a higher risk for overweight and obesity because of unhealthy diets, working shifts, long working hours, stress in the workplace, poor access to healthy food and low physical activity levels (Gupta & Gaur 2016).

### Sample selection

All 265 professional nurses, staff nurses and auxiliary nurses working at the private hospital in PMB on the days of data collection were invited to participate in the study (Yegambaram 2021). This was a convenience sample and was not representative of all nurses in PMB. Pregnant nurses were excluded from the study as it involved assessment of BMI, which is unreliable in pregnant women because of weight gain and oedema during pregnancy (Fakier, Petro & Fawcus 2017). Seven nurses were pregnant at the time of the study and were excluded, resulting in a total accessible population of 258 nurses. The statistician used Cochrane’s formula to determine that the minimum sample size required was 171 nurses (Bartlett, Kotrlik & Higgins 2001). To ensure representativity in terms of seniority of rank, the statistician determined that the sample should consist of 99 professional nurses, 43 staff nurses and 28 auxiliary nurses (Yegambaram 2021).

### Self-administered questionnaire

The researcher developed a self-administered questionnaire from literature in English to collect data. The self-administered questionnaire consisted of four sections. Section A collected data on demographic characteristics, section B covered lifestyle characteristics, section C collected data on body image and weight and section D covered eating habits. Only data related to BMI are reported here. The study supervisor and statistician determined face validity of the self-administered questionnaire. They checked that the questionnaire answered the study objectives, that the questions flowed logically and that no questions were leading, ambiguous or confusing. The pilot study was conducted using 10 professional nurses from the same private hospital. The pilot study participants were conveniently sampled on the day of the pilot study. They did not participate in the main study. Pilot study participants were given an information document and consent form to sign before data were collected from them. Because the participants did not report any problems with understanding the questions in the questionnaire, it was not revised for the main study. It took each participant between 10 min and 15 min to answer the questionnaire (Yegambaram 2021).

### Data collection

Data were collected in February 2020 on the premises of the private hospital in PMB. Nurses working both the day (07:00 to 19:00) and night shifts (19:00 to 07:00) were invited by the researcher to participate in the study. Nurses who agreed to participate in the study were given an information document to read and a consent form to sign before data were collected from them. Data were collected from one nurse at a time using a private room in each ward. The nurses answered the questionnaire first, and thereafter, the researcher took the weight, height and WC measurements from each participant. It took between 20 min and 25 min to collect data from each participant (Yegambaram 2021).

Weight was measured in kilograms using a calibrated, digital scale (Seca 874; Seca GmbH, Hamburg, Germany). Participants removed heavy objects such as jackets, shoes, keys, belts, cell phones and wallets before being weighed. Weight was measured three times to the nearest 0.1 kg, and a mean weight measurement was calculated (Yegambaram 2021). Height was measured in metres using a portable stadiometer (Seca 213; Seca GmbH, Hamburg, Germany) with a vertical backboard and adjustable headboard. The researcher ensured that the head, back, heels and buttocks of the participants touched the vertical backboard of the stadiometer. The participant’s head was aligned in the Frankfort horizontal plane before the height measurement was taken. Height was measured three times to the nearest 0.1 cm, and a mean height measurement was calculated. Body mass index was calculated using the mean weight and height measurements from each participant and the equation of weight in kilograms divided by height in metres squared. Body mass index was calculated to the nearest 0.1 kg/m^2^ and classified using the WHO BMI classification (WHO 2021a). Participants removed all heavy clothing such as jackets and jerseys (WHO 2017) before WC measurements were taken. The uppermost lateral border of the right ilium was located and marked. Another line was drawn vertically using the mid-axillary line, which extends from the armpit to the torso. Waist circumference measurements were taken using a Seca 201 Ergonomic Circumference Measuring Tape (Seca GmbH, Hamburg, Germany). The measuring tape was placed horizontally at the measurement mark with the tape parallel to the floor, snug and not tight. Waist circumference measurements were taken three times to the nearest 0.1 cm, and a mean WC measurement was calculated (Yegambaram 2021). A WC of greater than 94 cm in men and 80 cm in women indicated an increased risk for metabolic complications, while a WC of greater than 102 cm in men and 88 cm in women, indicated a substantially increased risk for metabolic complications (WHO 2011).

### Data analysis

After the researcher entered the data onto a Microsoft Excel spreadsheet, the data entry was cross-checked for errors by a research assistant. Thereafter, a statistician analysed the data using SPSS Version 26.0 (IBM Corp, Armonk, NY, USA). Descriptive statistics including frequencies, means and standard deviations were used to present the data. The chi-square goodness-of-fit-test and the independent samples *t*-test were used to analyse the data. A *p*-value of < 0.05 was regarded as statistically significant (Yegambaram 2021).

### Ethical considerations

The University of KwaZulu-Natal (UKZN) Biomedical Research Ethics Committee (BREC) (Ref: BE431/19) granted the study full ethics approval. The private hospital group gave approval to conduct the study but requested that the hospital remain anonymous. Nurses were free to decide when they wanted to participate. They could participate during their shift with permission from the Unit Manager or after their shift had ended. All participants gave written, informed consent before data collection could begin. Participants were also informed that participation in the study was voluntary and that they could withdraw from the study at any time, without incurring any penalties. The names and surnames of participants were not recorded to ensure anonymity. All data were secured through the use of password protected files, with the password known only to the researchers. Because data were collected from one nurse at a time, their participation did not affect their work or the operations in the ward (Yegambaram 2021).

## Results

### Sample characteristics

Although the target sample size was 171 participants, only 130 nurses participated, resulting in a response rate of 76%. Demographic characteristics of participants are shown in Table 1. Most participants were between 30 years and 39 years old (37.7%; *n* = 49), female (91.5%; *n* = 119) and African (71.5%; *n* = 93). One third of participants (33.1%; *n* = 43) had one dependent, while 39.2% (*n* = 51) were single parents (Table 1) (Yegambaram 2021).

**TABLE 1:** Demographic characteristics of participants (Yegambaram 2021).

Characteristic	*n*	%
**Age (years) (*N* = 130)**		
19–29	30	23.1
30–39	49	37.7
40–49	37	28.5
50–59	10	7.7
≥ 60	4	3.1
**Gender (*N* = 130)**		
Male	11	8.5
Female	119	91.5
**Race (*N* = 130)**		
African	93	71.5
White people	10	7.7
Indian	21	16.2
Coloured	6	4.6
**Number of dependants (*n* = 127)†**		
0	24	18.5
1	43	33.1
2	36	27.7
3	14	10.8
4	8	6.2
5	1	0.8
> 5	1	0.8
**Single parent (*N* = 130)**		
Yes	51	39.2
No	79	60.8

*Source:* Yegambaram, L.B., 2021, ‘Anthropometric status and dietary habits of registered nurses, enrolled nurses and enrolled nursing auxiliaries working at a private hospital in Pietermaritzburg, KwaZulu-Natal’, M.Sc dissertation, Department of Dietetics and Human Nutrition, University of KwaZulu-Natal

† , Some participants did not answer.

Fifty-three participants (40.8%) were professional nurses, 48 (36.9%) were staff nurses, three (2.3%) were midwives, 25 (19.2%) were auxiliary nurses and one participant (0.8%) was a clinical nurse specialist in the neonatal intensive care unit (NICU) (Table 2). Just over 35% (*n* = 46) of participants were qualified for 7–11 years, while 36.9% (*n* = 48) had been working as a nurse for 1–6 years. More than half the participants (*n* = 74) worked the day shift. Of those that worked the day shift, 53.1% (*n* = 69) worked a 12-h shift from 07:00 to 19:00. Just over 53% (*n* = 69) worked for five days a week. Most participants were off duty for two (36.9%; *n* = 48) to three (36.2%; *n* = 47) days a week (Table 2) (Yegambaram 2021).

**TABLE 2:** Work-related characteristics of participants (*N* = 130) (Yegambaram 2021).

Characteristic	*n*	%
**Nursing category**		
Professional nurse	53	40.8
Staff nurse	48	36.9
Midwife	3	2.3
Auxiliary nurse	25	19.2
Clinical nurse specialist (ICU)	1	0.8
**Number of years qualified**		
˂ 1	2	1.5
1–6	42	32.3
7–11	46	35.4
12–16	12	9.2
17–20	7	5.4
˃ 20	21	16.2
**Number of years working as a nurse**		
˂ 1	6	4.6
1–6	48	36.9
7–11	37	28.5
12–16	11	8.5
17–20	8	6.2
˃ 20	20	15.4
**Shift worked**		
Day	74	56.9
Night	56	43.1
**Working hours**		
07:00–19:00	69	53.1
19:00–7:00	56	43.1
07:00–16:00	5	3.8
**Number of days on duty per week**		
1	2	1.5
2	12	9.2
3	21	16.2
4	26	20.0
5	69	53.1
**Number of days off duty per week**		
2	48	36.9
3	47	36.2
4	16	12.3
5	17	13.1
6	2	1.5

*Source:* Yegambaram, L.B., 2021, ‘Anthropometric status and dietary habits of registered nurses, enrolled nurses and enrolled nursing auxiliaries working at a private hospital in Pietermaritzburg, KwaZulu-Natal’, M.Sc dissertation, Department of Dietetics and Human Nutrition, University of KwaZulu-Natal

ICU, intensive care unit.

Table 3 shows that 35.4% (*n* = 46) of participants were diagnosed with a medical condition by a medical doctor. Hypertension (11.5%; *n* = 15) was the most common medical condition, followed by diabetes (10%; *n* = 13). Other diagnosed medical conditions included anaemia, asthma, diverticulitis, epilepsy, human immunodeficiency virus (HIV), hyperthyroidism, insulin resistance, depression, hypothyroidism, polycystic ovarian syndrome (PCOS), irritable bowel syndrome (IBS), Meniere’s disease, migraines, psoriasis, reflux, sinusitis and tachycardia. A significant number of the participants reported that they did not smoke (82.3%; *n* = 107) (*p* = 0.000) and did not consume alcohol (59.2%; *n* = 77) (*p* = 0.035). Exactly half the sample (50%; *n* = 65) indicated that they did intentional exercise. For the purpose of this study, intentional exercise was defined as exercise that was planned and done with the intention of being physically active (Yegambaram 2021).

**TABLE 3:** Lifestyle characteristics of participants (*N* = 130) (Yegambaram 2021).

Characteristic	*n*	%	*p* †
**Diagnosed by a medical doctor with a chronic medical condition**			
Yes	46	35.4	> 0.05
No	84	64.6	> 0.05
**Medical condition**			
Diabetes	13	10.0	> 0.05
Hypertension	15	11.5	> 0.05
Heart disease	2	1.5	> 0.05
High cholesterol	4	3.1	> 0.05
Renal failure	0	-	> 0.05
Cancer	1	0.8	> 0.05
Other	23	17.7	> 0.05
**Participants who took chronic medication daily**			
Yes	35	26.9	> 0.05
No	11	8.5	> 0.05
**Smoking**			
Yes	23	17.7	**< 0.05**
No	107	82.3	**-**
**Alcohol consumption**			
Yes	53	40.8	**0.035**
No	77	59.2	-
**Participants did intentional exercise**			
Yes	65	50.0	1.000
No	65	50.0	-

*Source:* Yegambaram, L.B., 2021, ‘Anthropometric status and dietary habits of registered nurses, enrolled nurses and enrolled nursing auxiliaries working at a private hospital in Pietermaritzburg, KwaZulu-Natal’, M.Sc dissertation, Department of Dietetics and Human Nutrition, University of KwaZulu-Natal

Note: *p*-values in bold are statistically significant.

† , Chi-square goodness-of-fit test.

### Anthropometric characteristics

Just over 25% (*n* = 33) of the sample were overweight (*p* = 0.000), while 29.2% (*n* = 38) (*p* = 0.000), 13.1% (*n* = 17) and 18.5% (*n* = 24) were classified as obese class I, II and III, respectively. According to an independent samples *t*-test, females had a significantly higher mean BMI (33.55 kg/m^2^) compared with males (28.08 kg/m^2^) (*p* = 0.043) (Table 4) (Yegambaram 2021).

**TABLE 4:** Anthropometric characteristics of participants (*N* = 130) (Yegambaram 2021).

Characteristics	Total (*N* = 130)				Male (*n* = 11)				Female (*n* = 119)				*p*
	*n*	%	Mean	s.d.	*n*	%	Mean	s.d.	*n*	%	Mean	s.d.	
Weight (kg)	-	-	84.7	23.1	-	-	80.6	12.2	-	-	85.1	23.9	**0.043** †
Height (m)	-	-	1.6	0.1	-	-	1.7	0.1	-	-	1.6	0.1	-
BMI (kg/m^2^)	-	-	33.10	8.58	-	-	28.08	5.22	-	-	33.55	8.69	-
**BMI classification**													
Underweight	2	1.5	-	-	0	-	-	-	2	1.5	-	-	**< 0.05** ‡
Normal weight	16	12.3	-	-	3	2.3	-	-	13	10.0	-	-	-
Overweight	33	25.4	-	-	3	2.3	-	-	30	23.1	-	-	-
Obese class I	38	29.2	-	-	4	3.1	-	-	34	26.2	-	-	-
Obese class II	17	13.1	-	-	1	0.8	-	-	16	12.3	-	-	-
Obese class III	24	18.5	-	-	0	-	-	-	24	18.5	-	-	-

*Source:* Yegambaram, L.B., 2021, ‘Anthropometric status and dietary habits of registered nurses, enrolled nurses and enrolled nursing auxiliaries working at a private hospital in Pietermaritzburg, KwaZulu-Natal’, M.Sc dissertation, Department of Dietetics and Human Nutrition, University of KwaZulu-Natal

Note: *p*-values in bold are statistically significant.

s.d., standard deviation; BMI, body mass index.

† , Independent samples *t*-test;

‡ , Chi-square goodness-of-fit test.

An equal number of males (3.1%; *n* = 4) had a WC less than or equal to 94 cm and greater than 94 cm, respectively. Three males (2.3%) had a WC greater than 102 cm, indicating a substantially increased risk for metabolic complications. Eighty-six females (66.2%) had a WC greater than 88 cm, indicating a substantially increased risk for metabolic complications (Table 5) (Yegambaram 2021).

**TABLE 5:** Waist circumference measurements of participants (*N* = 130) (Yegambaram 2021).

Waist circumference	*n*	%
**Males (*n* = 11)**		
≤ 94 cm	4	3.1
> 94 cm	4	3.1
> 102 cm	3	2.3
**Females (*n* = 119)**		
≤ 80 cm	14	10.8
> 80 cm	19	14.6
> 88 cm	86	66.2

*Source:* Yegambaram, L.B., 2021, ‘Anthropometric status and dietary habits of registered nurses, enrolled nurses and enrolled nursing auxiliaries working at a private hospital in Pietermaritzburg, KwaZulu-Natal’, M.Sc dissertation, Department of Dietetics and Human Nutrition, University of KwaZulu-Natal

### Factors associated with a high body mass index

An independent samples *t*-test showed that the mean BMI of non-smokers (33.84 kg/m^2^) was significantly higher than that of smokers (29.58 kg/m^2^) (*p* = 0.030) (Table 6). The mean BMI of those who skipped meals (33.75 kg/m^2^) was significantly higher than for those who did not skip meals (29.63 kg/m^2^) (*p* = 0.005). Participants who skipped supper had a significantly higher BMI (36.27 kg/m^2^) than those who did not skip supper (32.00 kg/m^2^) (*p* = 0.013). There were no significant associations between any other lifestyle factors, demographic or work-related characteristics and BMI (Yegambaram 2021).

**TABLE 6:** Factors associated with a high body mass index among participants (Yegambaram 2021).

Factors	BMI (kg/m^2^)		*p* †
	Mean	s.d.	
**Gender**			
Male	28.08	5.22	**0.043**
Female	33.55	8.69	-
**Race**			
African	33.17	7.04	≥ 0.05
White people	36.59	13.88	-
Indian	29.92	6.82	-
Coloured	37.06	19.20	-
**Shift worked**			
Day	33.43	9.31	**≥ 0.05**
Night	32.64	7.56	-
**Diagnosed by a medical doctor with a chronic medical condition**			
Yes	35.07	9.90	≥ 0.05
No	32.04	7.65	-
**Smoking**			
Yes	29.58	10.16	**0.030**
No	33.84	8.05	-
**Alcohol consumption**			
Yes	32.75	9.12	≥ 0.05
No	33.32	8.23	-
**Participants did intentional exercise**			
Yes	33.88	8.29	≥ 0.05
No	32.30	8.85	-
**Meals skipped**			
Yes	33.75	8.96	**0.005**
No	29.63	5.11	-
**Supper skipped**			
Yes	36.27	9.83	**0.013**
No	32.00	7.87	-

*Source:* Yegambaram, L.B., 2021, ‘Anthropometric status and dietary habits of registered nurses, enrolled nurses and enrolled nursing auxiliaries working at a private hospital in Pietermaritzburg, KwaZulu-Natal’, M.Sc dissertation, Department of Dietetics and Human Nutrition, University of KwaZulu-Natal

Note: *p*-values in bold are statistically significant.

BMI, body mass index; s.d., standard deviation.

† , Independent samples *t*-test.

## Discussion

This study aimed to determine the anthropometric status of nurses working at a private hospital in PMB, KZN and to identify the factors related to a high BMI. Most nurses who participated in the study were between 30 and 39 years old and were female and African (Yegambaram 2021). The fact that the majority of nurses in this study were female is in line with the total number of female nurses on register (60 645) in KZN, which far outweighs the total number of male nurses on register (7292) (South African Nursing Council [SANC] 2021). The nurses on register include registered categories (professional nurses), enrolled nurses (staff nurses) and nursing auxiliaries (staff nurses). The total number of female nurses on register in SA is 8-fold greater than that of males (SANC 2021).

A high prevalence of overweight and obesity was found among the participants in the current study. The mean BMI of female participants in the current study was significantly higher than that of males (Yegambaram 2021). This is in line with findings of the SANHANES-1, which found that South African females had a higher mean BMI than males (Shisana et al. 2013). The prevalence of obesity among females in the current study (62.2%) was higher than that reported for South African females in the SANHANES-1 (39.2%) (Shisana et al. 2013) and the SADHS (41%) (NDoH et al. 2019). The high prevalence of obesity among female nurses in the current study is a cause for concern and requires urgent attention (Yegambaram 2021). The prevalence of obesity found in the current study is similar to the findings of Goon et al. (2013) in SA; however, a lower prevalence of obesity was found among nurses in other countries (Chin, Nam & Lee 2016; Kyle et al. 2017). This suggests that nurses in SA are facing a higher burden of obesity compared with nurses internationally (Yegambaram 2021).

In the current study, 66.2% of female participants had a WC greater than 88 cm. However, this is lower than another South African study on nurses, which found that just over 91% of female nurses were abdominally obese (Monakali et al. 2018). This finding is of concern as the risk of incident CVD increases in men and women with elevated WC. A 1 cm increase in WC is associated with a 2% increase in risk of future CVD (De Koning et al. 2007). Although BMI is well accepted as a useful predictor of overall and CVD mortality, WC is more strongly associated with cardiovascular events, even after adjustment for other risk factors (Piché et al. 2018). Given the fact that abdominal obesity alters CVD risk factors, WC should be routinely measured in clinical practice. Waist circumference is also useful in measuring the reduction in CVD risk after the adoption of healthy behaviours (Ross et al. 2020).

Despite the high prevalence of overweight and obesity found in the current study, most participants reported that they had not been diagnosed with any chronic medical conditions (Yegambaram 2021). This was an unexpected finding (Yegambaram 2021) as overweight and obesity are strong predictors for NCDs such as diabetes, CVD, musculoskeletal disorders and some cancers (WHO 2021a). This finding could be because of the relatively young age of the sample. Although they have not yet been diagnosed with any chronic medical conditions, they are at increased risk for developing these conditions as they age. It is also possible that participants in the current study were not aware of their medical conditions as they may have not sought medical attention when ill and may not have had regular health assessments (Yegambaram 2021). Bana et al. (2016), who investigated the healthcare seeking behaviour of healthcare professionals in Pakistan, found that the majority of nurses (74%) had not visited a doctor for any reason in over 12 months. Almost all nurses (99.7%) in the Pakistani study indicated that they self-medicated when they were ill. The most common reason for nurses not seeking healthcare was the high cost of healthcare. This is also a possible reason why nurses in the current study were unaware of their medical conditions (Yegambaram 2021). Healthcare professionals need to be encouraged to have regular health assessments, to visit the doctor on time and be aware of the dangers of self-medicating (Bana et al. 2016).

The current study found that the mean BMI of smokers (29.58 kg/m^2^) was significantly lower than the BMI of non-smokers (33.84 kg/m^2^) (Yegambaram 2021). Smoking cigarettes that contain nicotine increase the resting metabolic rate; this together with a reduced intake of food may lead to weight loss. Reduced food intake with smoking could also result from cigarettes being a behavioural alternative to eating (Audrain-McGovern & Benowitz 2011). In the current study, the mean BMI of participants who skipped supper was significantly higher than those who ate supper. A study by Yamamoto et al. (2021) found that skipping dinner was significantly associated with overweight and obesity. Skipping dinner was found to have a stronger association with weight gain and overweight and obesity than skipping breakfast. A possible mechanism for skipping dinner and weight gain may be an excessive energy intake because of an increased appetite after skipping dinner. Encouraging regular consumption of dinner may be a useful recommendation in reducing the risk for overweight and obesity (Yamamoto et al. 2021).

## Study limitations and recommendations

Although all private hospitals in PMB were invited to participate in the study, only one private hospital participated. The sample was therefore not a true representation of all nurses working in private hospitals in PMB, which prevents generalised conclusions from being drawn. In addition, there was a poor response rate in this study because of the nurses not being willing to participate. No biochemical tests or medical tests were conducted in the study to screen for undiagnosed existing NCDs, because of financial constraints. In addition, no body composition analyses were conducted to determine body fat percentage and the contribution of muscle and fat to body weight. This would have provided a more accurate indication of anthropometric status. This study highlights the need for more in-depth assessment of the anthropometric status of nurses. A larger study including all nurses in SA should be conducted and participants should be recruited from both the private and public sectors (Yegambaram 2021).

## Conclusion

The majority of nurses who participated in this study were overweight or obese. Waist circumference measurements revealed that most nurses had an increased risk for metabolic complications. However, most participants were not diagnosed with an NCD. Obesity and high WC were more of a problem among female nurses, compared with males. Factors associated with a high BMI included being female, not smoking and skipping meals, particularly supper. The high prevalence of overweight and obesity and the increased risk for metabolic complications among the nurses in this study are a concern and require urgent intervention.
